# Elevated cell-free mitochondria DNA level of patients with premature ovarian insufficiency

**DOI:** 10.1186/s12884-023-05769-1

**Published:** 2023-06-22

**Authors:** Xing-Yu Zhou, Yi-Zhen Yang, Jun Zhang, Xiao-Fei Zhang, Yu-Dong Liu, Zhe Wang, Shi-Ling Chen

**Affiliations:** Center for Reproductive Medicine, Department of Gynecology and Obstetrics, Nanfang Hospital, Southern Medical University, No. 1838 Guangzhou Northern Road, Guangzhou, 510515 Guangdong China

**Keywords:** Premature ovarian insufficiency, Mitochondria, Cell-free mitochondria DNA, Inflammation

## Abstract

**Background:**

Premature ovarian insufficiency (POI) patients present with a chronic inflammatory state. Cell-free mitochondria DNA (cf-mtDNA) has been explored as a reliable biomarker for estimating the inflammation-related disorders, however, the cf-mtDNA levels in POI patients have never been measured. Therefore, in the presenting study, we aimed to evaluate the levels of cf-mtDNA in plasma and follicular fluid (FF) of POI patients and to determine a potential role of cf-mtDNA in predicting the disease progress and pregnancy outcomes.

**Methods:**

We collected plasma and FF samples from POI patients, biochemical POI (bPOI) patients and control women. Quantitative real-time PCR was used to measure the ratio of mitochondrial genome to nuclear genome of cf-DNAs extracted from the plasma and FF samples.

**Results:**

The plasma cf-mtDNA levels, including COX3, CYB, ND1 and mtDNA79, were significantly higher in overt POI patients than those in bPOI patients or control women. The plasma cf-mtDNA levels were weakly correlated with ovarian reserve, and could not be improved by regular hormone replacement therapy. The levels of cf-mtDNA in FF, rather than those in plasma, exhibited the potential to predict the pregnancy outcomes, although they were comparable among overt POI, bPOI and control groups.

**Conclusions:**

The increased plasma cf-mtDNA levels in overt POI patients indicated its role in the progress of POI and the FF cf-mtDNA content may hold the value in predicting pregnancy outcomes of POI patients.

**Supplementary Information:**

The online version contains supplementary material available at 10.1186/s12884-023-05769-1.

## Introduction

Premature ovarian insufficiency (POI) is a life-changing disease for women, which is associated with the progressive loss of ovarian activity prior to age 40, a decade before natural menopause, resulting in an increased risk of infertility and long-term health. For early detection and intervention, the European Society for Human Reproduction and Embryology adjusted the only laboratory diagnostic indicator for POI from a follicle-stimulating hormone (FSH) value of 40 mIU/mL to 25 mIU/mL [1]. Ovarian reserve decline is thought to be a continuous process, and POI has been described as encompassing the spectrum of ovarian dysfunction from decreased fecundity (occult POI), and elevated FSH (biochemical POI, bPOI) to eventually amenorrhea (overt POI) [2]. FSH is the only current laboratory diagnostic indicator for diagnosing POI, but its inter and intra-cycle variability has significantly limited its assessment of POI progression [3]. Anti-Müllerian hormone (AMH) is reported as a more sensitive marker for characterizing ovarian reserve and has been applied to the diagnosis of POI [4], but there is still no consensus on its diagnostic threshold [5]. It is noted that infertility is an earlier manifestation of POI and usually occurs in the years leading up to amenorrhea. However, the commonly used ovarian reserve makers, such as AMH, could not predict the fertility outcomes of women with diminished ovarian reserve very well [6]. Therefore, it is still necessary to search for biomarkers with high sensitivity and specificity the prediction of POI progresses and assessment of POI women’s fecundity.

Cell-free mitochondria DNA (cf-mtDNA) is known to be released sequentially from injured mitochondria and then from cells [7, 8]. The extracellular mtDNAs are mainly released by apoptotic or necrotic cells, and are generally involved in cellular inflammatory response and intercellular communication [9]. Another active source of cf-mtDNA is the extracellular vesicles, which maintain mitochondrial function by establishing cell-to-cell communication in response to the pro-inflammatory stimuli [10]. Under some certain pathological conditions, large amounts of mtDNA are placed into extracellular fluid and bloodstream, triggering inflammatory responses [9]. As lacking for protective histones and series of DNA repair mechanisms, mtDNA exhibits a sensitive property to external stress, which determines the sufficient detection amount of cf-mtDNA. In addition, the property of resistance to nuclease degradation ensures the integrity of cf-mtDNA fragments as much as possible [11]. Together, cf-mtDNA has been explored as a reliable biomarker for estimating the inflammation-related disorders. Accumulating evidences demonstrated that extracellular fluid cf-mtDNA levels differ significantly between diseased individuals and healthy controls, and have the potential to be the biomarkers indicating disease onset and development including cancers [12], infectious diseases [13], cardiovascular diseases [14], neurodegenerative diseases [15] and mental disorders [16]. According to our recent [17, 18] and previous studies [19, 20], POI patients also present with a chronic inflammatory state. Despite evidence for a link between POI and cf-mtDNA, the cf-mtDNA levels in POI patients have never been measured, which prompts our interest in developing cf-mtDNA as a potential biomarker for POI.

Therefore, in the presenting study, we aimed to evaluate the levels of cf-mtDNA in plasma and follicular fluid (FF) of patients with POI and to determine a potential role of cf-mtDNA in predicting the disease progress and in vitro fertilization (IVF) outcomes.

## Materials and methods

This study was approved by the ethics committee of Nanfang Hospital (NFEC-2017–197). All enrolled patients were fully informed of the purpose of the study and signed informed consent.

### Criteria for study subjects

**Fig. 1:**
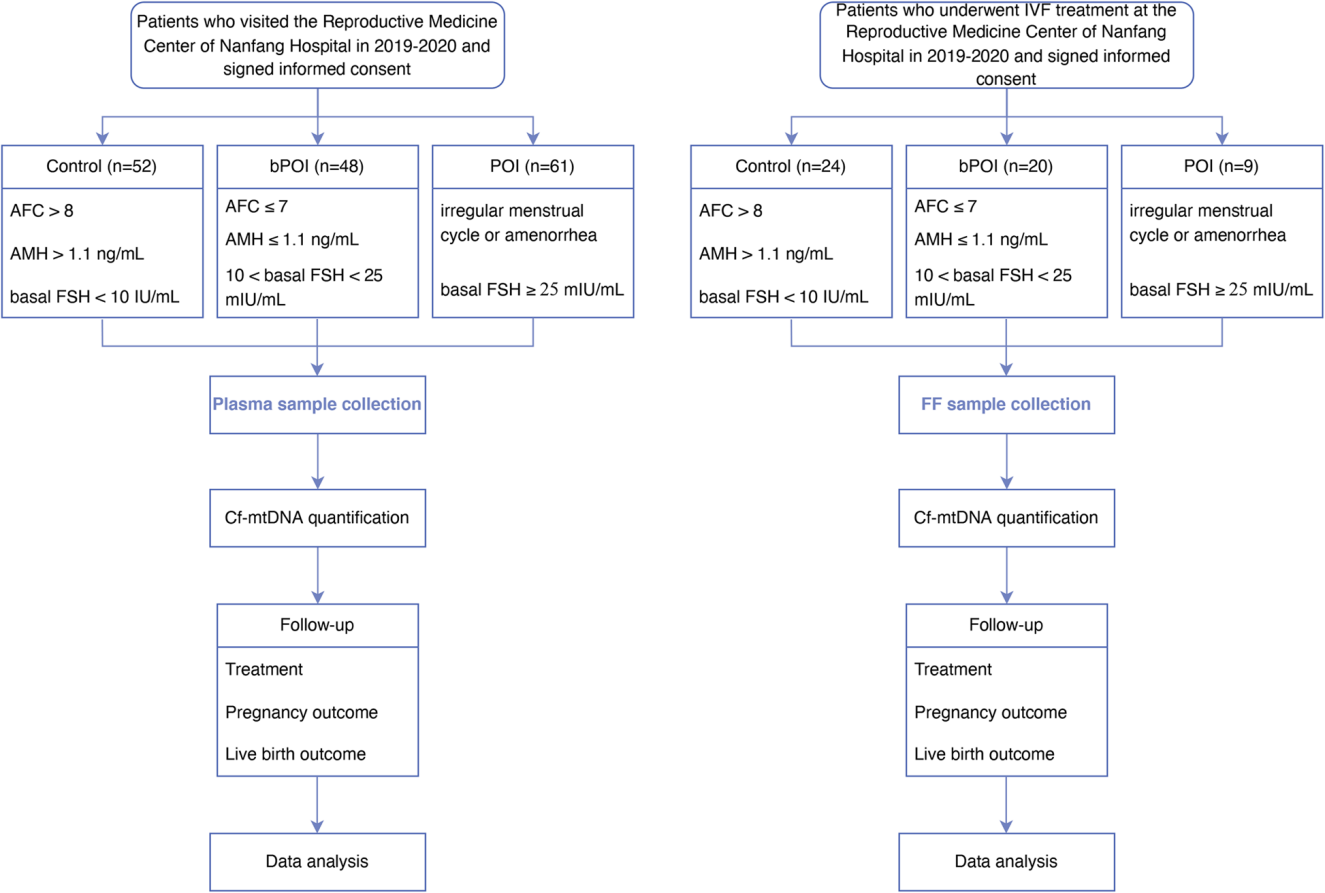
Flow chart

This study selected a subset of patients who were treated at the Reproductive Medicine Center of Nanfang Hospital from 2019 to 2020. The plasma samples of newly diagnosed 61 POI patients, 48 bPOI patients and 52 control women were obtained. Among infertile women who had received an IVF treatment, we obtained FF samples from 9 POI patients, 20 bPOI patients and 24 control women (Fig. 1).

The inclusion criteria for POI were as follows: (1) irregular menstrual cycle (< 24 days or > 35 days) or amenorrhea for at least 4 months, and (2) an elevated FSH level ≥ 25 mIU/mL on two occasions ≥ 4 weeks apart. Patients with regular menstrual cycle who also met one of the following criteria at least were regarded as bPOI: (1) antral follicle count (AFC) ≤ 7; (2) AMH ≤ 1.1 ng/mL; (3) 10 < basal FSH < 25 mIU/mL [21, 22]. The control group was women with severe male factor infertility or tubal/cervical factor infertility. Women in control group should have regular menstrual cycles (25–35 days), normal ovarian morphology and ovarian reserve (basal FSH < 10 IU/mL, bilateral AFC > 8, and AMH concentrations > 1.1 ng/mL). The patients’ ages were all below 40 years.

Patients with X chromosomal abnormality or genetic mutations, iatrogenic ovarian injury (e.g., ovarian or pelvic surgery, radiotherapy and chemotherapy), autoimmune diseases (e.g., Hashimoto’s thyroiditis, systemic lupus erythematosus and Sjogren’s syndrome), as well as other reproductive endocrine diseases (e.g., polycystic ovary syndrome and endometriosis) were all excluded from our study.

A total of 30 POI patients were treated with regular hormone replacement therapy (HRT). The duration of HRT ranged from 3 months to 8 years, with a median duration of 8 month. Among them, 23.3% (7/30) of patients took estradiol valerate 2 mg + cyproterone acetate 1 mg, 53.3% (16/30) took estradiol 2 mg + dydrogesterone 10 mg, 13.3% (4/30) took estradiol valerate 2 mg + progesterone 50 mg/100 mg, and 10.0% (3/30) took ethinylestradiol 30 µg + drospirenone 3 mg/ desogestrel 15µg .

### IVF outcomes

In our center, the conventional ovarian stimulation regimens, included gonadotropin-releasing hormone (GnRH) antagonist or GnRH agonist long protocol, were used depending on individual circumstance. Ovaries were monitored, and at the time when at least one follicle reached 18 mm in diameter, a dose of 5000–10,000 IU human chorionic gonadotropin (hCG) in combination with 0.2 mg GnRH agonist was administered to trigger final oocyte maturation in the GnRH antagonist cycle, while a dose of 5000–10,000 IU hCG was used alone in the long GnRH agonist cycle. After 36 h, the oocytes were retrieved via transvaginal ultrasound-guided aspiration. For POI and bPOI patients with serious poor ovarian response, it was typical of us to apply the conventional GnRH antagonist protocol in the cycle of the first attempt. If no developing follicles were observed, we would switch to a natural cycle regimen. When follicles reached a diameter of larger than 16 mm, a dose of 5000 IU hCG was administered, and the oocyte pick-up were performed after 36 h.

The oocytes were assessed for quality and maturity under the microscope and were further categorized as germinal vesicle (GV), metaphase I (MI), and metaphase II (MII) with respect to the maturity stage. The quality of the day 3 embryos and blastocytes was recorded by the morphological criteria [23, 24]. After embryo or blastocyst transfer, clinical pregnancy was determined by observation of the gestational sac on ultrasound in the following 4 weeks. The pregnancy and live birth rates we calculated were referred to the cumulative pregnancy rate and the cumulative live birth rate, which were defined as the number of pregnancies and deliveries resulting from one aspiration IVF cycle, including all cycles in which fresh and/or frozen embryos were transferred until one pregnancy/delivery occurred or all embryos were used.

### Sample collection and preparation

For newly diagnosed patients with informed consent, their whole blood (10 mL) was drawn from a peripheral vein into an Ethylene Diamine Tetraacetic Acid (EDTA) anticoagulant vacuum blood collection tube. Within 2 h of collection, the blood was centrifuged at 2000 rpm and 4 °C for 10 min. The plasma was then removed and transferred into sterile 1.5 ml tubes. Aliquoted plasma was centrifuged again at 13,000 rpm and 4 °C for 10 min, with the supernatant being aliquoted into a new 1.5 mL tube and stored at -80 °C until further use.

For each patient undergoing an IVF treatment with informed consent, the follicles were aspirated without flushing and blood contamination on the day of oocyte retrieval. To prevent the FF samples from being contaminated by blood, we collected 2 mL of FF only from the first dominant follicle with a diameter of over 16–18 mm, and all FFs centrifuged at 2000 rpm and 4 °C for 10 min, and then immediately stored at -80 °C until cf-mtDNA quantification.

### Cf-mtDNA extraction and quantification

A volume of 200 μL plasma or FF was processed for cf-DNA extraction by Serum/Plasma Circulating DNA Kit (Tiangen, Beijing, China), according to the manufacturer’s instructions. The extracted cf-DNAs were eluted in 40 μL of elution buffer and stored at -40 °C for further use in cf-mtDNA relative quantification. The mitochondrial genome to nuclear genome ratio (Mt/N), assessed by quantitative real-time PCR (qRT-PCR), is often used to reflect changes in the mtDNA content. For cf-mtDNA quantification, primers of COX3, CYB, ND1, mtDNA79 and mtDNA230 (as mitochondrial DNA genes), and 18S rRNA (as nuclear reference gene) were synthesized by Sangon Biotech company (Supplementary Table 1, Shanghai, China) [12]. To evaluate the ratio of cf-mtDNA/cf-nDNA, qRT-PCR was performed. Briefly, 2 μL of extracted cf-DNA, 0.8 μL of 10 μM primers (forward and reverse), 7.2 μL diethylpyrocarbonate (DEPC)-treated water and 10 μL of 2 × TB Green Premix Ex Taq II (Takara, Dalian, China) were mixed. Cycling conditions were as follows: 95 °C for 30 s, then 40 cycles of 95 °C for 5 s and 60 °C for 20 s, 95 °C for 5 s again and 60 °C for 60 s, then 50 °C for 30 s. The plasma/FF cf-mtDNA relative copy number was calculated by 2^−ΔΔCT^ equation.

### Statistical analysis

Statistical analyses were performed with SPSS (version 26.0) and R (version 1.4.1106). Continuous variable data was presented as the mean ± standard deviation (SD) for its normal distribution or median (interquartile range) when the normal distribution was absent. The Kolmogorov–Smirnov test and Levene’s test were used successively to test the normal distribution and homogeneity of variances. When data conformed to the normal distribution and equal variance, the one-way ANOVA test was used for the comparison between multiple groups, and the Least-Significant Difference (LSD) was used for the post-hoc comparison. Otherwise, the Kruskal–Wallis test and the Bonferroni method were used for testing and post-hoc comparison, respectively.

The associations between the levels of plasma cf-mtDNA and ovarian reserve were analyzed by Spearman correlation analysis. Receiver Operating Characteristic (ROC) curve was drawn to estimate the predictive value of some indicators for the pregnancy and live birth outcomes. A *P* value < 0.05 was considered as statistically significant.

## Results

### Patients’ baseline characteristics

In this case–control study, a total of 161 plasma samples (52 in the control group, 48 in the bPOI group and 61 in the POI group) and 53 FF samples (24 in the control group, 20 in the bPOI group and 9 in the POI group) were collected, respectively. The patients’ baseline characteristics were successively recorded in Table 1 and Table 2.

**Table 1:** Baseline characteristics of patients with plasma specimens

	**Control** **(*n* = 52)**	**bPOI** **(*n* = 48)**	**POI** **(*n* = 61)**	***P***
Age (years)	30.00 (6.00)	33.00 (5.00)^**^	30.00 (6.00)^##^	< 0.001^a^
BMI (kg/m^2^)	21.80 (2.98)	20.65 (3.60)	20.81 (3.30)	0.073^a^
Infertility duration (years)	3.00 (3.00)	3.00 (4.00)	3.00 (4.00)	0.339^a^
AFC (n)	17.50 (11.00)	4.00 (4.00)^**^	1.00 (2.00)^**/##^	< 0.001^a^
AMH (ng/ml)	3.42 (2.11)	0.56 (0.75)^**^	0.06 (0.07) ^**/##^	< 0.001^a^
Basal FSH (mIU/mL)	6.30 (1.45)	8.64 (6.02)^*^	80.83 (44.17) ^**/##^	< 0.001^a^
Basal LH (mIU/mL)	4.74 (3.09)	4.99 (3.55)	37.97 (25.93) ^**/##^	< 0.001^a^
Basal E_2_ (pg/mL)	36.67 (22.61)	41.76 (32.75)	13.00 (17.67) ^**/##^	< 0.001^a^
N. patient performing IVF treatment, % (n)	100 (52/52)	100 (48/48)	11.48 (7/61)	/
Ovarian stimulation protocol, % (n)				< 0.001
GnRH agonist long protocol	34.6 (18/52)	12.5 (6/48)	0	
GnRH antagonist protocol	65.4 (34/52)	79.2 (38/48)	57.1% (4/7)	
Natural cycle	0	8.3 (4/48)	42.9% (3/7)	
Total dose of Gn	1862.5 (1547)	2325 (1425)	450 (1200) ^**/##^	< 0.001^a^
Clinical pregnancy rate, % (n)	80.4 (41/51)	26.1 (12/46)	14.3 (1/7)	< 0.001
Live birth rate, % (n)	72.5 (37/51)	21.7 (10/46)	0 (0/7)	< 0.001

*BMI* Body mass index (calculated as weight in kilograms divided by the square of height in meters), *AFC* Antral follicle count, *AMH* Anti-Müllerian hormone, *FSH* Follicle-stimulating hormone, *LH* Luteinizing hormone, *E*_*2*_ Estradiol, *IVF* In vitro fertilization

Median (interquartile range), calculated using SPSS (version 26.0)

^a^Kruskal-Wallis test

Bonferroni method for post-hoc comparison, *compared to group “Control”, ^#^ compared to group “bPOI”, ^*^P < 0.05, ^**^P < 0.01, ^##^*P* < 0.01

**Table 2:** Clinical characteristics of patients with follicular fluid specimens

	**Control** **(*n* = 24)**	**bPOI** **(*n* = 20)**	**POI** **(*n* = 9)**	***P***
Age (years)	33.50 (7.00)	34.50 (6.00)	33.00 (7.00)	0.487^a^
BMI (kg/m^2^)	22.02 ± 3.00	21.78 ± 2.60	20.53 ± 3.17	0.417^b^
Infertility duration (years)	3.00 (5.00)	3.00 (4.30)	1.00 (5.00)	0.309^a^
AFC (n)	15.00 (4.00)	5.00 (4.00)^**^	1.00 (2.00)^**/#^	< 0.001^a^
AMH (ng/mL)	3.79 (1.38)	0.43 (0.55)^**^	0.05 (0.12)^**/##^	< 0.001^a^
Basal FSH (mIU/mL)	6.77 ± 1.30	15.29 ± 4.18^**^	46.58 ± 24.61^**/##^	< 0.001^b^
Basal LH (mIU/mL)	5.01 (2.17)	6.26 (3.14)	11.79 (41.10) ^**/##^	< 0.001^a^
Basal E_2_ (pg/ml)	33.37 ± 11.31	31.56 ± 15.13	24.82 ± 10.65	0.240^b^
Ovarian stimulation protocol, %(n)				< 0.001
GnRH agonist long protocol	62.5 (15/24)	10 (2/20)	0	
GnRH antagonist protocol	37.5 (9/24)	90 (18/20)	66.7 (6/9)	
Natural cycle	0	0	33.3 (3/9)	
Total dose of Gn	1925 (1118.75)	2175 (1131.25)	600 (1762.5) ^*/#^	0.002^a^
Clinical pregnancy rate, %(n)	50 (12/24)	30 (6/20)	11.1 (1/9)	0.092
Live birth rate, %(n)	37.5 (9/24)	23.1 (3/20)	7.7 (1/9)	0.133

Median (interquartile range) or mean ± standard deviation (SD), calculated using SPSS (version 26.0)

*BMI* Body mass index (calculated as weight in kilograms divided by the square of height in meters), *AFC* Antral follicle count, *AMH* Anti-Müllerian hormone, *FSH* Follicle-stimulating hormone, *LH* Luteinizing hormone, *E*_*2*_ Estradiol

^a^Kruskal-Wallis test; ^b^one-way ANOVA test

Bonferroni or Least-Significant Difference (LSD) method for post-hoc comparison, *compared to group “Control”, ^#^ compared to group “bPOI”, **P < 0.01, ^#^*P* < 0.05, ^##^*P* < 0.01

As shown were the study of that part of plasma samples in Table 1, BMI and infertility years were both matched among the three groups (*P* = 0.073; *P* = 0.339). The age of patients in the control group and the POI group was comparable (*P* = 0.889, not recorded in the table). Moreover, the ovarian reserve (based on the AFC, AMH and FSH levels) was also assessed and the baseline hormonal status was almost evaluated in each patient at day 3 of the menstruation. Compared with the control group, the levels of AFC (*P* < 0.001) and AMH (*P* < 0.001) were significantly decreased in the bPOI group, while the level of FSH increased (*P* < 0.001). Then comparing the POI group with the bPOI group, the levels of AFC (*P* < 0.001) and AMH (*P* < 0.001) in the POI group were further decreased, while the FSH level increased furtherly (*P* < 0.001). Besides, the POI group had a higher luteinizing hormone (LH) level and a lower estradiol (E_2_) level than bPOI. Unfortunately, only few POI patients (11.48%) received an IVF treatment subsequently. The ovarian stimulation regimen was different among the control, bPOI and POI groups (*P* < 0.001). And as expected, pregnancy and live birth rates were significantly higher in the control group than in the patients with bPOI and POI (*P* < 0.05).

As for that part of FF samples, in Table 2, the age, BMI and infertility years were all equivalent among the three groups (*P* = 0.487; *P* = 0.417; *P* = 0.309). As predicted, the levels of AFC and AMH decreased gradually among the control, bPOI and POI groups, while the FSH level increased gradually, and all the differences were statistically significant (*P* < 0.05). The ovarian stimulation regimens were also different among the three group, but the difference in the pregnancy and live birth rate did not meet the statistically required criteria (*P* > 0.05).

### Comparison of plasma and FF cf-mtDNA levels among groups

**Fig. 2:**
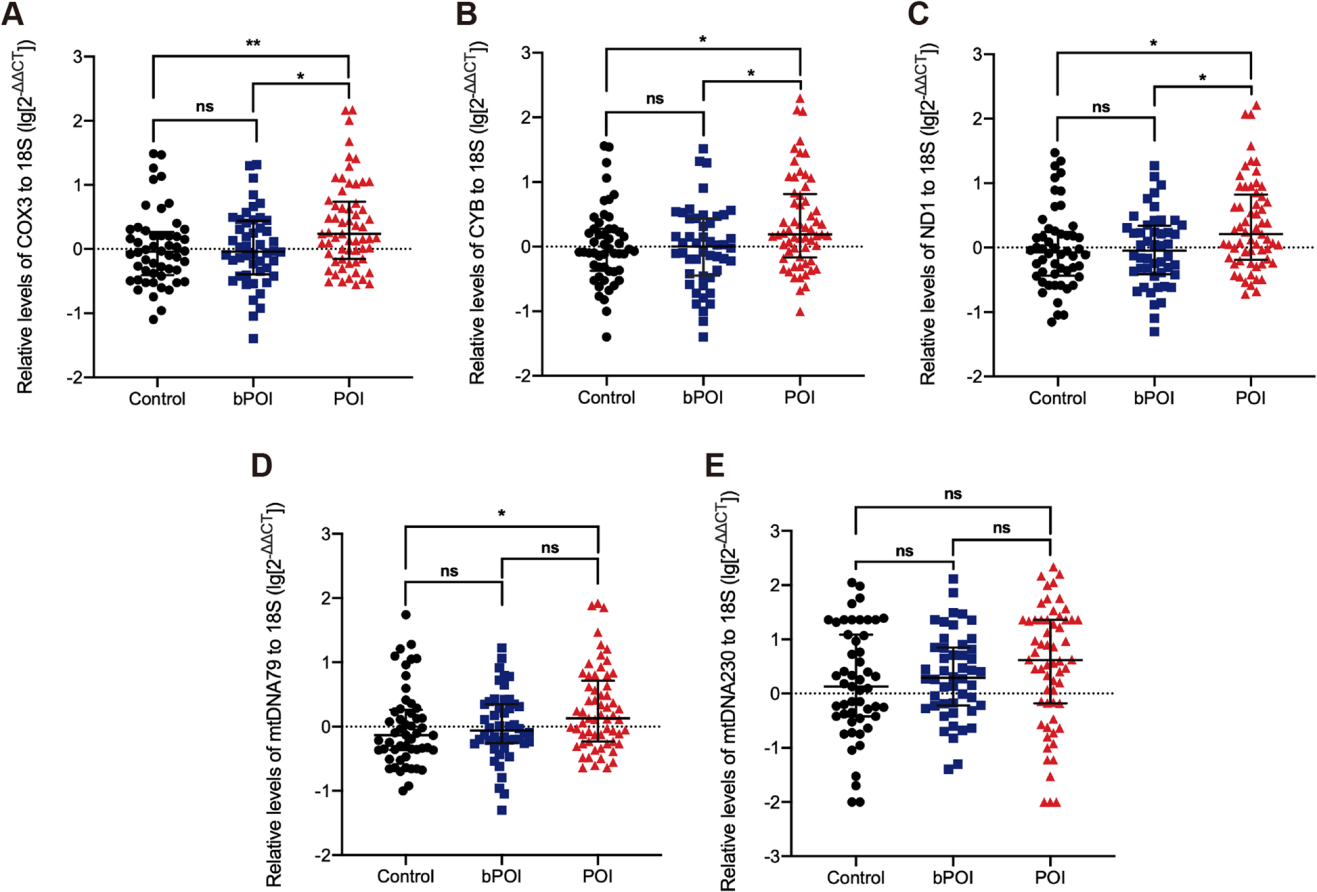
Comparison of the plasma COX3 levels (A), CYB levels (B), ND1 levels (C), mtDNA79 levels (D) and mtDNA230 levels (E) among control, bPOI and POI groups. Kruskal–Wallis test and Bonferroni method. ^*^P < 0.05, ^**^P < 0.01

**Fig. 3:**
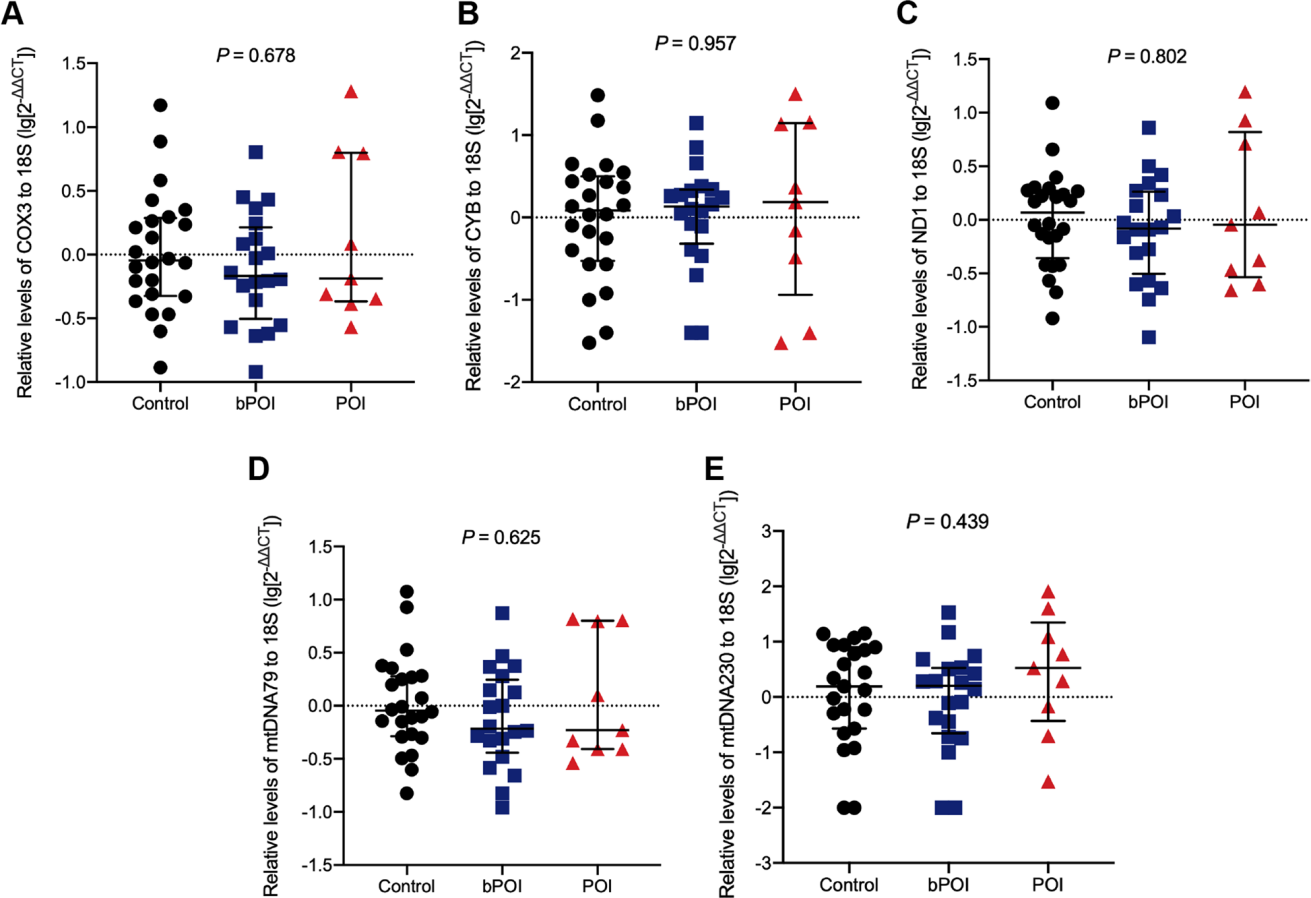
Comparison of FF COX3 levels (A), CYB levels (B), ND1 levels (C), mtDNA79 levels (D) and mtDNA230 levels (E) among control, bPOI and POI groups. Kruskal–Wallis test

Quantification by qRT-PCR of selected cf-mtDNA fragments in plasma and FF samples were displayed in Figs. 2 and 3. According to the distribution of the data calculated by 2^−ΔΔCT^ equation, we then performed a log10 calculation on the resulting data for compositional harmony. The expression levels of COX3 (*P* < 0.01), CYB (*P* < 0.05), ND1 (*P* < 0.05) and mtDNA79 in plasma (*P* < 0.05) were significantly increased in the POI group than in the control group. And in the comparison with the bPOI group, the POI group also had higher levels of plasma COX3 (*P* < 0.05), CYB (*P* < 0.05) and ND1 (*P* < 0.05). However, there was no difference in the plasma cf-mtDNA expression between the control group and the bPOI group (Fig. 2). Furthermore, we investigated whether the plasma cf-mtDNA levels differed in POI patients with or without regular HRT. However, there were no difference in the levels of plasma cf-mtDNA regardless of whether the patients were treated with regular HRT (Supplementary Fig. 1).

In Fig. 3, no significant differences were found in the FF cf-mtDNA expression between the three groups. Notably, the POI group only contained 9 FF samples, which was not suitable for comparison with the other two groups in the number of specimens.

### Correlation between the plasma cf-mtDNA level and the ovarian reserve

Given that the plasma cf-mtDNA levels differed among the above groups, we performed the Spearman correlation analyses to explore the relationship between cf-mtDNA level and the ovarian reserve characteristics. As shown in Table 3, the results indicated that the plasma cf-mtDNA levels were significantly and negatively correlated with AFC and AMH, while was positively correlated with FSH.

**Table 3:** Correlations between the plasma cf-mtDNA levels and ovarian reserve

	**COX3**		**CYB**		**ND1**		**mtDNA79**		**mtDNA230**	
	**r**	***P***	**r**	***P***	**r**	***P***	**r**	***P***	**r**	***P***
FSH	0.181	0.023	0.181	0.023	0.205	0.010	0.180	0.024	0.177	0.026
AFC	-0.211	0.009	-0.178	0.028	-0.146	0.072	-0.150	0.066	-0.124	0.128
AMH	-0.167	0.046	-0.132	0.117	-0.132	0.118	-0.136	0.107	-0.107	0.205

Spearman correlation analysis, calculated using SPSS (version 26.0)

*FSH* Follicle-stimulating hormone, *AFC* Antral follicle count, *AMH* Anti-Müllerian hormone

### Predictive value of the FF cf-mtDNA level for IVF outcomes

To evaluate the predictive value of cf-mtDNA level for the pregnancy and live birth rate, we first performed multiple ROC curve analyses using that part data of plasma samples, while the results were not very considerable (AUC < 0.7, Supplementary Table 2).

And then, we looked at the data of FF samples which were more representative of the local microenvironment of the ovary. As shown in Table 4, in the prediction of pregnancy and live birth outcomes by FF cf-mtDNA, the AUC were estimated at 0.761 (0.610–0.912) and 0.706 (0.544–0.868), respectively, while the AMH with the values of 0.622 (0.444–0.800) and 0.613 (0.416–0.809) (Table 4).

**Table 4:** Predictive value of the FF cf-mtDNA level for pregnancy and live birth outcomes

	**Valid cases (n)**	**cf-mtDNA**				**AMH**				***P***
		**AUC** **[95%CI]**	**Specificity (%)**	**Sensitivity (%)**	***P***	**AUC** **[95%CI]**	**Specificity (%)**	**Sensitivity (%)**	***P***	
Pregnancy	41	0.761 [0.610, 0.912]	81.80	68.40	0.004	0.622 [0.444, 0.800]	72.70	63.20	0.178	0.289
Live birth	41	0.706 [0.544, 0.868]	60.70	84.60	0.033	0.613 [0.416, 0.809]	71.40	69.20	0.245	0.500

AUC [95% CI]: area under the ROC curve [95% confidence interval]

ROC curve analysis, calculated using R (version 1.4.1106)

Furthermore, to assess whether the FF cf-mtDNA was an independent risk factor affecting the pregnancy and live birth outcomes, a multiple logistic regression analysis including age, BMI, AMH, FSH, and cf-mtDNA was conducted. The results suggested that high levels of FF cf-mtDNA predicted poor pregnancy (OR = 3.774, 95% CI: 1.520–15.320, *P* = 0.024) and live birth outcomes (OR = 3.540, 95% CI: 1.404–15.290, *P* = 0.033).

## Discussion

In this study, we delineated the plasma cf-mtDNA levels, including COX3, CYB, ND1 and mtDNA79, were significantly elevated in overt POI patients when compared to those in bPOI patients and control women. The plasma cf-mtDNA levels were weakly correlated with AFC and AMH, and could not be lowered by regular HRT. The levels of cf-mtDNA in FF, rather than those in plasma, exhibited the potential to predict the IVF outcomes, although they were equivalent among overt POI, bPOI and control groups.

Mitochondrial DNA is a double-stranded circular DNA molecule with 16,569 base pairs (bp) that encodes 13 protein subunits, 22 transfer RNAs, and 2 ribosomal RNAs. All proteins encoded by mitochondrial DNA were involved in the oxidative phosphorylation system, including Cytochrome oxidase (COX1, COX2, COX3, and Cytochrome b), NADH dehydrogenase (ND1, ND2, ND3, ND4, ND4L, ND5, and ND6), and ATP synthase (ATP6 and ATP8) [25]. The mtDNA 79 bp (mtDNA79) and 230 bp (mtDNA230) fragments was amplified form mitochondrial 16S rRNA gene [12]. In our study, COX3, CYB, ND1, mtDNA79, and mtDNA230 were selected to represent general cf-mtDNA levels in plasma and FF, due to their relatively high expression levels and superior performance in distinguishing patients from heathy controls [12, 13, 26, 27]. At the same time, 18S rRNA was chosen as a reference for cf-nDNA to eliminate or weaken the effects of concomitant cf-nDNA release [26, 27].Due to the covalently closed circular double-stranded structure with lack of histone protection and containing hypomethylated CpG motifs reminiscent of an ancestral bacterial origin, the released cf-mtDNA have been considered as a damage-associated molecular patterns (DAMPs) [28]. Accumulated studies have suggested that mtDNA exerts its proinflammatory effects through interaction with Toll-like receptor 9 (TLR9), which subsequently activates the downstream inflammatory pathways [7, 29, 30] and lead to a variety of inflammatory diseases [31]. Specific to the field of ovarian research, it was reported that cf-mtDNA had adverse effects on the oocyte quality and ovarian granulosa cell biosynthesis, via activating the inflammatory TLR9/NF-κB p65/MAPK p38 pathways [19]. Therefore, cf-mtDNA was considered to be a promising indicator for the studying the dynamic changes in inflammatory response.

As mentioned above, we found the high plasma cf-mtDNA levels in POI patients, reflecting the existence of excessive systemic inflammation, which was consistent with the recent findings, that is, inflamm-aging may be a new pathogenic mechanism of POI [20]. Liu P et al. revealed the imbalance of chemokine and growth factors in FF of bPOI patients, indicating the immune disturbance of local ovarian microenvironment [32], and their further study confirmed POI patients’ enhanced TH1 proinflammatory response in both periphery environment and ovarian microenvironment [33]. Oxidative stress was also recognized as a reflection of the inflammation, and POI patients exhibited excessive oxidative stress, manifesting the increase of neutrophil to lymphocyte ratio, total oxidant status, oxidative stress index level, and platelet-activating factor level [34, 35]. Similarly, we recently reported the plasma levels of advanced oxidation protein products (AOPP), a novel marker of oxidized proteins, that significantly elevated in POI patients, and represented a negative correlation with AMH/AFC [17]. Our recent metabolomics study also found that 18-HETE, a kind of oxylipins, was accumulated in the plasma of POI patients, which reflected the excessive lipid oxidation in POI [18]. Then, combining with the results of this study, the increase of plasma cf-mtDNA levels provided a further verification on the excessive inflammation in POI. Furthermore, the bPOI was defined as the early stage of POI showing a slightly elevated FSH level, but no difference of cf-mtDNA content both in plasma and FF between bPOI and control groups were observed in our results. It should be noted here that some part of bPOI patents will be in a state of diminished ovarian reserve (DOR), which refers to a decline in the number and quality of oocytes without strict age limits and diagnostic criteria, for a long time, and we cannot distinguish the bPOI from the DOR patients in clinic by far [36]. It could be one of the reasons why plasma cf-mtDNA levels did not differ between the bPOI and control groups. Another reason may be that the release and elimination of cf-mtDNA can still keep at a state of dynamic equilibrium in the bodies of bPOI patients. Combined with the negative correlation between the cf-mtDNA level and ovarian reserve status, we proposed that the plasma cf-mtDNA content may be a potential biomarker for monitoring the development of POI. Based on this reasoning, we furtherly investigated whether HRT could reduce the plasma cf-mtDNA levels, but found that the regular HRT did not influence it. This result was contrary to the previous cognition, that is, estrogen deficiency leads to various clinical manifestations of POI. And then, applying HRT was supposed to have some feedback on many indicators. It is possible that the increase of cf-mtDNA in POI itself has nothing to do with the role of hormones, but is driven by some proinflammatory factors, such as the elevated basal FSH levels [37].

In this study, there was no significant difference in FF cf-mtDNA levels among the control group, bPOI group and POI group, but the cf-mtDNA in FF demonstrated its potential to predict the IVF outcomes. It is important to noted that FF is a better indicator of ovarian microenvironment than plasma [38], however, it is difficult to obtain FF samples from the overt POI patients because of their follicle depletion. We have only collected 9 FF samples from overt POI patients, and the small sample size may restrict the effectiveness of comparison between POI and control group. In addition to the limitation of FF sample size, another reason may be that the dominant follicles of POI patients can still maintain the oxidative-antioxidant homeostasis, as the FF samples collected in this study were derived from the dominant follicles.

Infertility is the earliest symptom in POI, which usually occurs a few years before the onset of amenorrhea. Therefore, some experts put forward the concepts of occult POI and biochemical POI to define the early stages of this disease, according to the declined fecundity and the elevated FSH levels. For those patients, it is generally believed that the spontaneous pregnancy rate is very low and the effect of IVF treatment is also not satisfactory [39]. But excitingly, some studies have demonstrated the efficacy of ovulation induction and subsequent IVF treatment [40]. However, some reliable biomarkers are still lacking to predict the pregnancy outcomes after the IVF treatment. Serum AMH was proved to be an available indicator to evaluate ovarian reserve and predict stimulation response, but could not well reflect the oocyte quality or pregnancy opportunity [41]. Up to now, some studies have shown that cf-mtDNA in the spent embryo culture medium, derived from the oocytes and embryos, was correlated with the oocyte maturation, embryo morphological features, and blastulation [42–45]. Recently, the association between cf-mtDNA in FF and oocyte developmental competence has been revealed [46, 47], and cf-mtDNA in FF has been selected as a promising biomarker to predict the IVF outcomes in women without reproductive disorders [48]. Consistently, our study found that cf-mtDNA in FF, rather than it in plasma, could effectively predict the pregnancy outcomes of bPOI and POI patients. Moreover, cf-mtDNA in FF showed a superior prediction efficiency than AMH. FF is composed of plasma components and secretions produced by peripheral granulosa cells and oocytes [38, 49], so mtDNA released by apoptotic granulosa cells and poor-quality oocytes preferentially enters into the FF. To sum up our results and previous studies, FF cf-mtDNA appears to be a potential biomarker for the IVF outcome, although some studies with larger sample size are still required to confirm its applicability in POI patients.

In summary, significantly increased plasma cf-mtDNA levels were observed in patients with overt POI compared with those in bPOIs and controls. Although this finding has limited significance for clinical practice, it indicates that cf-mtDNA may predict the progress of POI. Further studies should focus on the role of FF cf-mtDNA in embryo development and whether cf-mtDNA could be considered as a marker for pregnancy prediction in ovarian disorders like POI.

## Supplementary Information

**Additional file 1:** **SupplementaryTable 1. **PCR primer sequences of mtDNAs. **Supplementary Table 2. **Predictive value of the plasma cf-mtDNA level forpregnancy and live birth outcomes. **Supplementary Figure 1. **Comparisonof the plasma cf-mtDNA levels between regular HRT and irregular HRT groups inPOI patients. Mann-Whitney test. 

## Data Availability

The datasets used and/or analysed during the current study are available from the corresponding author on reasonable request.
