# Supplementary material for: Elevated cell-free mitochondria DNA level of patients with premature ovarian insufficiency
Source: BMC Pregnancy Childbirth. 2023 Jun 22;23:462. doi: 10.1186/s12884-023-05769-1 (PMC10288775; doi:10.1186/s12884-023-05769-1)
Supplement: Supplementary file 1 — Additional file 1: SupplementaryTable 1. PCR primer sequences of mtDNAs. Supplementary Table 2. Predictive value of the plasma cf-mtDNA level forpregnancy and live birth outcomes. Supplementary Figure 1. Comparisonof the plasma cf-mtDNA levels between regular HRT and irregular HRT groups inPOI patients. Mann-Whitney test. [file 12884_2023_5769_MOESM1_ESM.docx]

**Supplementary Table 1. PCR primer sequences of mtDNAs.**

| **Gene** | **Primer sequences** | |
| --- | --- | --- |
| 18S rRNA | Forward | AGAAACGGCTACCACATCCA |
|  | Reverse | CCCTCCAATGGATCCTCGTT |
| COX3 | Forward | ATGACCCACCAATCACATGC |
|  | Reverse | ATCACATGGCTAGGCCGGAG |
| CYB | Forward | ACATCGGCATTATCCTCCTG |
|  | Reverse | GTGTGAGGGTGGGACTGTCT |
| ND1 | Forward | ATACCCATGGCCAACCTCCT |
|  | Reverse | GGGCCTTTGCGTAGTTGTAT |
| mtDNA79 | Forward | AGCCGCTATTAAAGGTTCG |
|  | Reverse | CCTGGATTACTCCGGTCTGA |
| mtDNA230 | Forward | AGCCGCTATTAAAGGTTCG |
|  | Reverse | GGGCTCTGCCATCTTAACAA |

**Supplementary Table 2 Predictive value of the plasma cf-mtDNA level for pregnancy and live birth outcomes.**

|  | **Valid cases (n)** | **cf-mtDNA** | | | | |
| --- | --- | --- | --- | --- | --- | --- |
|  |  | **AUC [95%CI]** | **Specificity (%)** | **Sensitivity (%)** | ***P*** |  |
| Pregnancy | 104 | 0.630 [0.522, 0.737] | 70.00 | 55.60 | 0.022 |  |
| Live birth | 104 | 0.608 [0.499, 0.718] | 52.60 | 70.20 | 0.057 |  |

AUC [95% CI]: area under the ROC curve [95% confidence interval].

ROC curve analysis, calculated using R (version 1.4.1106).


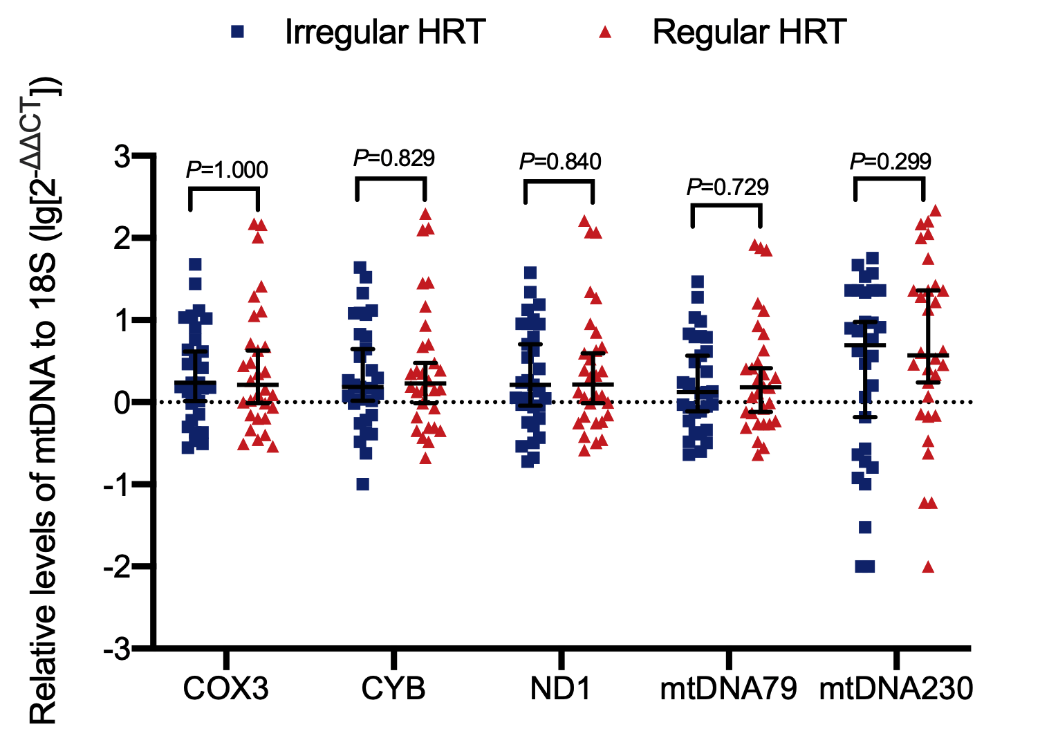


**Supplementary Figure 1** Comparison of the plasma cf-mtDNA levels between regular HRT and irregular HRT groups in POI patients. Mann-Whitney test.
